# Computational modeling of the effects of autophagy on amyloid-β peptide levels

**DOI:** 10.1186/s12976-020-00119-6

**Published:** 2020-02-26

**Authors:** Kyungreem Han, Soon Ho Kim, MooYoung Choi

**Affiliations:** 1 Laboratory of Computational Biology, National Heart, Lung and Blood Institute, National Institutes of Health, Bethesda, MD USA; 2 Department of Physics and Astronomy and Center for Theoretical Physics, Seoul National University, Seoul, South Korea

**Keywords:** Autophagy model, Amyloid-β peptide, Alzheimer’s disease

## Abstract

**Background:**

Autophagy is an evolutionarily conserved intracellular process that is used for delivering proteins and organelles to the lysosome for degradation. For decades, autophagy has been speculated to regulate amyloid-β peptide (Aβ) accumulation, which is involved in Alzheimer’s disease (AD); however, specific autophagic effects on the Aβ kinetics only have begun to be explored.

**Results:**

We develop a mathematical model for autophagy with respect to Aβ kinetics and perform simulations to understand the quantitative relationship between Aβ levels and autophagy activity. In the case of an abnormal increase in the Aβ generation, the degradation, secretion, and clearance rates of Aβ are significantly changed, leading to increased levels of Aβ. When the autophagic Aβ degradation is defective in addition to the increased Aβ generation, the Aβ-regulation failure is accompanied by elevated concentrations of autophagosome and autolysosome, which may further clog neurons.

**Conclusions:**

The model predicts that modulations of different steps of the autophagy pathway (i.e., Aβ sequestration, autophagosome maturation, and intralysosomal hydrolysis) have significant step-specific and combined effects on the Aβ levels and thus suggests therapeutic and preventive implications of autophagy in AD.

## Introduction

Autophagy (from the Greek, *autos*, which means “self”, and *phagein*, “to eat”) is an evolutionarily conserved catabolic pathway, which delivers cytoplasmic constituents such as proteins and organelles to the lysosome for degradation and recycling [1–3]. Autophagy regulates protein quality, energy balance, and metabolic homeostasis, and furthermore it plays a role in the decision-making of cellular life and death, depending on the context of its activation [2–5]. The energy molecules and metabolic building blocks such as adenosine triphosphate (ATP) and amino acids, respectively, which are the recycled products of autophagy, regulate the consecutive steps of the autophagy process, i.e., sequestration (or autophagosome formation), autophagosome maturation (autolysosome formation), and intralysosomal hydrolysis, via mammalian target of rapamycin (mTOR) (for amino acids) and AMP-activated protein kinase (AMPK) pathways (for ATP) [6–9].

Neurons are especially vulnerable to autophagy dysfunction because they rely heavily upon autophagy for preventing the accumulation of toxic substances such as damaged proteins and protein aggregates [10–12]. For this, the brain is considered to be the most severely affected organ by the autophagy dysfunction [11, 12]: It is particularly related to the development of neurodegenerative disorders such as Alzheimer’s disease (AD) and Parkinson’s disease (PD) [10, 11, 13–17]. In young (healthy) neurons, autophagy can efficiently deliver the toxic substances along the unusually large architectures of axons and dendrites to lysosomes, which are concentrated in the cell body, while old (deteriorated) neurons have reduced autophagic degradation efficacy. It is becoming increasingly evident that the autophagic degradations of aggregate-prone proteins in neurons are highly substrate-selective [18]. These selective pathways appear to rely on the specific interactions between substrates and autophagy receptors/adaptors to sequester certain substrates within autophagosomes. Then the substrates proceed to the same degradation machinery as non-selective (bulk) autophagy [19–22]. Furthermore, it has been suggested that modulation of substrate–receptor/adaptor interactions can be considered as a new therapeutic strategy for neurodegenerative disorders [18].

AD, a common form of dementia, is one of the most prevalent neurological disorders associated with aging as its incidence is rapidly growing every year [23, 24]. The neuropathological hallmarks include deposition of extracellular plaques and formation of intracellular neurofibrillary tangles (NFTs). The plaques and NFTs predominantly consist of amyloid-β peptides (Aβ) and tau proteins, respectively. According to the amyloid hypothesis, an accumulation of Aβ is the primary factor for the onset and progression of AD and the rest of the process including the NFT formation is the secondary effects of the Aβ toxicity [25–27]. An increased intracellular Aβ level is observed prior to the onset of extracellular plaque formation.

Aβ consists of 36 to 43 amino acids and is intracellularly generated by specific proteolytic cleavage of the amyloid precursor protein (APP), an integral membrane protein which is concentrated in the synapses of neurons. An altered balance between generation, degradation, secretion (from the intra to the extracellular space of a neuron), and clearance (from the extracellular space) of Aβ is responsible for the intracellular accumulation and extracellular plaque formation. It has been reported that the Aβ generation rate is abnormally high in the early and late stages of AD [28]. Aβ is degraded preferentially via autophagy; yet during late stages of AD autophagosomes fail to fuse with lysosomes [28]. In addition, the Aβ secretion rate depends on the autophagy activity [29–31]: the secretion rate is reduced in mice lacking autophagy-related gene 7 (Atg7) [30]. On the other hand, the autophagic activity is influenced by the intracellular Aβ concentration [28, 32–34]. The Aβ clearance rate in the extracellular space varies with the Aβ concentration in a biphasic manner [35]. The AD patient is associated with a decrease in clearance by roughly 30%, which may lead to toxic levels of Aβ accumulation in the extracellular space over about 10 years [36].

Although many individual mechanisms have been studied for decades, the association of Aβ kinetics with autophagy activity and the roles of autophagy in the pathogenesis of AD remain elusive. In this study, we develop a mathematical model for autophagy with respect to Aβ kinetics, integrating various individual molecular and cellular data sets, in hope of providing a unified framework for understanding the complex dynamics between autophagy and Aβ pathways. Simulations are performed to identify the quantitative relationship between autophagy activity and Aβ kinetics, including the intra and extracellular levels, secretion, clearance, and autophagic degradation. This may provide a starting point for understanding the effects of autophagy on the pathogenesis of AD and implications of pharmacological autophagy modulation for AD therapy and prevention.

### Mathematical model

**Fig. 1:**
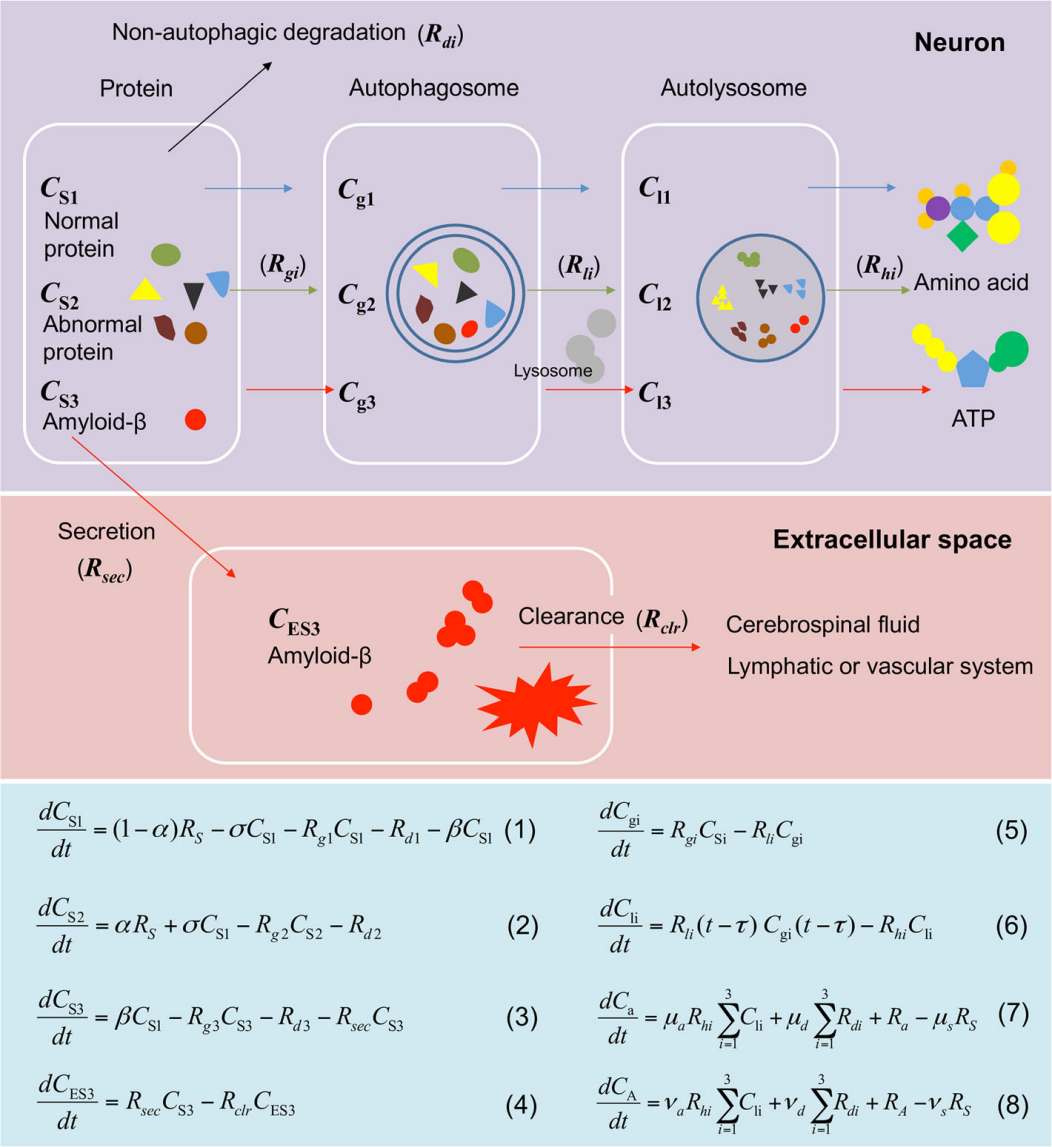
Schematic diagram of the model system. The rounded rectangles with white borders illustrate four compartments: 1) intracellular protein, 2) autophagosome, 3) autolysosome, and 4) extracellular amyloid-β (Aβ) peptide. *C*_S1_, *C*_S2_, and *C*_S3_ denote the concentrations of intracellular resident protein S1, abnormal protein S2, and amyloid-β peptide S3, respectively. *C*_gi_ and *C*_li_ represent the concentrations of autophagosomes and autolysosomes, respectively, from Si (i = 1, 2, 3). *C*_ES3_ stands for the extracellular Aβ concentration. *R*_*gi*_, *R*_*li*_, *R*_*hi*_, and *R*_*di*_ are the specific rates of autophagosome formation, autolysosome formation, intralysosomal hydrolysis, and non-autophagic degradation, respectively, for Si (i = 1, 2, and 3 again). *R*_*sec*_ and *R*_*clr*_ denote respectively the rates of Aβ secretion and clearance. The differential equations describe variations of the concentrations of proteins (Eqs. (1)–(4)), autophagosomes (Eq. (5)), autolysosomes (Eq. (6)), amino acids (Eq. (7)), and ATP (Eq. (8))

The model assumes a four-compartment description of the autophagy process, including 1) intracellular protein (including normal/abnormal protein and intracellular Aβ), 2) autophagosome, 3) autolysosome, and 4) extracellular Aβ compartments (Fig. 1).

#### Dynamic equations

Intracellular proteins are classified as resident proteins S1 which conduct normal functions in a cell, abnormal proteins S2 including damaged proteins and those abnormally transcribed or translated, and amyloid-β peptide S3. We write the equations for the dynamics of concentrations *C*_S1_, *C*_S2_, and *C*_S3_ of S1, S2, and S3, respectively, in the form:

1 $$ \frac{d{C}_{\mathrm{S}1}}{dt}=\left(1-\alpha \right){R}_S-\sigma {C}_{\mathrm{S}1}-{R}_{g1}{C}_{\mathrm{S}1}-{R}_{d1}-\beta {C}_{\mathrm{S}1}, $$

2 $$ \frac{d{C}_{\mathrm{S}2}}{dt}=\alpha {R}_S+\sigma {C}_{\mathrm{S}1}-{R}_{g2}{C}_{\mathrm{S}2}-{R}_{d2}, $$

3 $$ \frac{d{C}_{\mathrm{S}3}}{dt}=\beta {C}_{\mathrm{S}1}-{R}_{g3}{C}_{\mathrm{S}3}-{R}_{d3}-{R}_{sec}{C}_{\mathrm{S}3}, $$

where *R*_*S*_ represents the (total) protein synthesis rate (from DNA) and *α* is the fraction of S2, namely, S1 and S2 are produced at the rates of (1 – *α*)*R*_*S*_ and *αR*_*S*_, respectively. *σ* is the rate constant for deterioration of S1 (i.e., transformation from S1 to S2). *R*_*gi*_ and *R*_*di*_ represent the specific rates of autophagosome formation and the non-autophagic degradation of Si (for i = 1, 2, and 3), respectively. *β* denotes the rate constant for Aβ generation and *R*_*sec*_ is the Aβ secretion specific rate from the intra to the extracellular space.

The dynamics of the Aβ concentration in the extracellular space *C*_ES3_ reads:

4 $$ \frac{d{C}_{\mathrm{ES}3}}{dt}={R}_{sec}{C}_{\mathrm{S}3}-{R}_{clr}{C}_{\mathrm{ES}3}, $$

where *R*_*clr*_ denotes the specific clearance rate for Aβ in the extracellular space.

Variations of the intracellular autophagosome concentration with time are determined by the difference between the autophagosome formation specific rate *R*_*gi*_ and the autolysosome formation specific rate *R*_*li*_ (*i* = 1, 2, and 3 for S1, S2, and S3, respectively). With *C*_gi_ denoting the concentration of autophagosome originating from Si (i = 1, 2, and 3), the dynamics of the concentration is governed by the following equation:

5 $$ \frac{d{C}_{\mathrm{gi}}}{dt}={R}_{gi}{C}_{\mathrm{Si}}-{R}_{li}{C}_{\mathrm{gi}}. $$

The intracellular concentration *C*_li_ of autolysosomes originating from Si (i = 1, 2, and 3) is determined by the difference between *R*_*li*_ and the intralysosomal hydrolysis specific rate *R*_*hi*_ (*i* = 1, 2, and 3). The equation governing the dynamics takes the form:

6 $$ \frac{d{C}_{\mathrm{li}}}{dt}={R}_{li}\left(t-\tau \right)\;{C}_{\mathrm{gi}}\left(t-\tau \right)-{R}_{hi}{C}_{\mathrm{li}}. $$

Note that the autolysosome concentration at time *t* is affected by the autophagosome concentration at time *t* – *τ*, earlier by the delay time *τ*, which is taken to be 8 min (*τ* = 480 s) [37–39].

The dynamics of intracellular amino acids, the concentration of which is denoted by *C*_a_ reads:

7 $$ \frac{d{C}_{\mathrm{a}}}{dt}={\mu}_a{R}_{hi}\sum \limits_{i=1}^3{C}_{\mathrm{li}}+{\mu}_d\sum \limits_{i=1}^3{R}_{di}+{R}_a-{\mu}_s{R}_S. $$

The first and second terms on the right-hand side correspond to the supply of amino acids due to the autophagic intralysosomal hydrolysis and non-autophagic protein degradation, respectively, with appropriate constants *μ*_*a*_ and *μ*_*d*_ describing the average numbers of amino acids produced from autophagic and non-autophagic degradation, respectively. The third term represents the rate of amino acid supply from extracellular fluid into cells that is assumed to be proportional to the metabolic demand (i.e., protein synthesis rate *R*_*S*_) and the loss of protein (i.e., secretion rate of Aβ, given by *R*_*sec*_*C*_S3_) such that *R*_*a*_ = *μ*_*c*_*R*_*S*_ + *μ*_*β*_*R*_*sec*_*C*_S3_ with appropriate constants *μ*_*c*_ and *μ*_*β*_. The last term describes the reduction of amino acids due to protein synthesis with the constant *μ*_*s*_, the average number of amino acids in a protein molecule.

The dynamic equation for intracellular ATP concentration *C*_A_ reads:

8 $$ \frac{d{C}_{\mathrm{A}}}{dt}={v}_a{R}_{hi}\sum \limits_{i=1}^3{C}_{\mathrm{li}}+{v}_d\sum \limits_{i=1}^3{R}_{di}+{R}_A-{v}_s{R}_S $$

where *ν*_*a*_ and *ν*_*d*_ are the average numbers of ATP molecules produced from autophagic degradation and from non-autophagic degradation, respectively. The net intracellular ATP generation rate *R*_*A*_ is assumed to be *R*_*A*_ = *ν*_*c*_*R*_*S*_ + *ν*_*β*_*R*_*sec*_*C*_S3_ that is associated with the metabolic demand and the loss of protein, with appropriate constants *ν*_*c*_ and *ν*_*β*_. The last term corresponds to the reduction of ATP due to protein synthesis, where *ν*_*s*_ gives the average number of ATP molecules in a protein.

n average protein molecule in a cell is assumed to be composed of 500 amino acid residues; in other words, 500 amino acids are consumed in unit protein synthesis (i.e., *μ*_*s*_ = 500). Considering that elongation of one amino acid during translation requires approximately four ATP molecules, we have assumed that 2000 ATP molecules are required for the synthesis of a protein (*ν*_*s*_ = 2000). However, the numbers of amino acids and ATP molecules per degradation of one protein via autophagic or non-autophagic protein degradation have been set to be less than those required in the protein synthesis, because the efficacy of protein recycling is expected to be less than 100%; this yields *μ*_*a*_ = *μ*_*d*_ = *μ*_*β*_ = *ν*_*a*_ = *ν*_*d*_ = *ν*_*β*_ = 300, *μ*_*c*_ = 200, and *ν*_*c*_ = 1700.

Details of the autophagy-related rates in Eqs. (1) to (8) are given in the following subsections. The parameters are summarized in Table 1.

**Table 1:** Parameters in computer simulations

Parameter	Value	Unit	Description
${r}_{gi}^{(0)}$	1.12 × 10^−5^	s^−1^	Rate constant for autophagosome formation of Si (i = 1, 2, 3) (normal value)
*α*	1.00 × 10^−2^	(unitless)	Fraction of S2 in protein synthesis rate *R*_*S*_
*β*^(0)^	5.56 × 10^−10^	s^−1^	Rate constant for Aβ generation (normal value)
*σ*	4.00 × 10^−7^	s^−1^	Rate constant for deterioration of S1
*ω*_*g*_	−9.43 × 10^−1^	mM^−0.1^	Constant for autophagosome formation (Aβ dependency)
*ζ*_*g*_	1.00 × 10^−1^	(unitless)	Constant for autophagosome formation (Aβ dependency)
*ψ*_*g*_	1.01 × 10^2^	mM^−1^	Constant for autophagosome formation (Aβ dependency)
*k*_*g*_	2.83	mM	Constant for autophagosome formation (ATP dependency)
*p*_*g*_	3.00	mM	Constant for autophagosome formation (ATP dependency)
*a*_*g*_	4.50	mM	Constant for autophagosome formation (amino acids dependency)
*γ*_*g*_	1.22	(unitless)	Constant for autophagosome formation (amino acids dependency)
*ξ*_*g*_	7.49 × 10^−2^	mM^−1^	Constant for autophagosome formation (amino acids dependency)
${r}_{li}^{(0)}$	2.47 × 10^−5^	s^−1^	Rate constant for autolysosome formation of Si (i = 1, 2, 3) (normal value)
*k*_*l*_	2.83	mM	Constant for autolysosome formation (ATP dependency)
*p*_*l*_	3.00	mM	Constant for autolysosome formation (ATP dependency)
${r}_{hi}^{(0)}$	1.39 × 10^−5^	s^−1^	Rate constant for intralysosomal hydrolysis of Si (i = 1, 2, 3) (normal value)
*δ*_*h*_	7.24 × 10^−1^	(unitless)	Exponent for intralysosomal hydrolysis (ATP dependency)
*k*_*h*_	2.99	mM	Constant for intralysosomal hydrolysis (ATP dependency)
*r*_*s*_	1.48 × 10^−5^	mM ⋅ s^−1^	Rate constant for protein/organelle synthesis
*k*_*s*_	1.77 × 10^1^	mM	Constant for protein/organelle synthesis (amino acids dependency)
${C}_{\mathrm{A}}^{(m)}$	3.00	mM	ATP concentration corresponding to maximal protein/organelle synthesis rate
*r*_sec_	4.67 × 10^−9^	s^−1^	Rate constant for Aβ secretion
*r*_*clr*_	2.23 × 10^−1^	mM^−1^ ⋅ s^−1^	Rate constant for Aβ clearance
*ω*_*ext*_	6.34 × 10^−5^	mM	Rate constant for Aβ clearance

#### Autophagosome formation

We take the autophagosome formation specific rates *R*_*gi*_ from Si (for i = 1, 2, and 3), which depend on the intracellular concentrations *C*_S3_ of Aβ [28, 32–34], *C*_A_ of ATP [40, 41], and *C*_a_ of amino acids [42] as follows:

9 $$ {R}_{g1}\left({C}_{\mathrm{S}3},{C}_{\mathrm{a}},{C}_{\mathrm{A}}\right)={r}_{g1}\left({\omega}_g{C_{\mathrm{S}3}}^{\zeta_g}+{\psi}_g{C}_{\mathrm{S}3}+1\right)\frac{{C_{\mathrm{A}}}^4}{{C_{\mathrm{A}}}^4+{k_g}^4}\frac{{p_g}^{12}}{{C_{\mathrm{A}}}^{12}+{p_g}^{12}}\frac{{a_g}^8}{{C_{\mathrm{a}}}^8+{a_g}^8}\left(1+{\gamma}_g{e}^{-{\xi}_g{C}_{\mathrm{a}}}\right), $$

10 $$ {R}_{g2}\left({C}_{\mathrm{S}3},{C}_{\mathrm{a}},{C}_{\mathrm{A}}\right)={r}_{g2}\left({\omega}_g{C_{\mathrm{S}3}}^{\zeta_g}+{\psi}_g{C}_{\mathrm{S}3}+1\right)\frac{{C_{\mathrm{A}}}^4}{{C_{\mathrm{A}}}^4+{k_g}^4}\frac{{p_g}^{12}}{{C_{\mathrm{A}}}^{12}+{p_g}^{12}}\left(1+{\gamma}_g{e}^{-{\xi}_g{C}_{\mathrm{a}}}\right), $$

11 $$ {R}_{\mathrm{g}3}\left({C}_{\mathrm{S}3},{C}_{\mathrm{a}},{C}_{\mathrm{A}}\right)={r}_{g3}\left({\omega}_g{C_{\mathrm{S}3}}^{\zeta_g}+{\psi}_g{C}_{\mathrm{S}3}+1\right)\frac{{C_{\mathrm{A}}}^4}{{C_{\mathrm{A}}}^4+{k_g}^4}\frac{{p_g}^{12}}{{C_{\mathrm{A}}}^{12}+{p_g}^{12}}\left(1+{\gamma}_g{e}^{-{\xi}_g{C}_{\mathrm{a}}}\right), $$

where *r*_*gi*_ is the rate constant for autophagosome formation from Si (for i = 1, 2, and 3), with appropriate constants *ω*_*g*_, *ζ*_*g*_, *ψ*_*g*_ (for Aβ), *k*_*g*_, *p*_*g*_ (ATP), *a*_*g*_, *γ*_*g*_, and *ξ*_*g*_ (amino acids).

Intracellular Aβ affects the mTOR signaling, which negatively regulates autophagy induction, exhibiting a nonlinear relationship: The mTOR activity increases (i.e., suppressing autophagosome formation) with the Aβ level until reaching a certain threshold (~ 0.5 μM) and then the activity gradually decreases (restoring autophagosome formation) above the threshold concentration [28, 32–34]. This nonlinear relationship has been included in Eqs. (9)–(11) as a simple algebraic equation in the form of ${\omega}_g{C_{\mathrm{S}3}}^{\zeta_g}+{\psi}_g{C}_{\mathrm{S}3}+1$.

The remaining part of the right-hand side contains the ATP and amino acid dependency of the autophagosome formation step. Under normal conditions, it appears that S2 and S3, abnormal proteins and Aβ, are preferentially degraded by autophagy. However, as the intracellular energy/nutrient reduces due to, e.g., starvation or increased metabolic demand, all the proteins (S1, S2 and S3) are degraded non-selectively for the rapid supply of essential energy molecules (e.g., ATP) and metabolic building blocks (i.e., amino acids) [21, 22, 43, 44]. Therefore, it is assumed in this model that the autophagosome formation rate from resident proteins S1, which is lower than that from abnormal proteins and Aβ (S2 and S3) under normal conditions, becomes gradually equal to those of S2 and S3 as the amino acid concentration is decreased [45–48].

#### Autolysosome formation and intralysosomal hydrolysis

The autolysosome formation specific rate *R*_*li*_ reads (*i* = 1, 2, and 3 for S1, S2, and S3)

12 $$ {R}_{li}\left({C}_{\mathrm{A}}\right)={r}_{li}\frac{{C_{\mathrm{A}}}^4}{{C_{\mathrm{A}}}^4+{k_l}^4}\frac{{p_l}^{12}}{{C_{\mathrm{A}}}^{12}+{p_l}^{12}}, $$

where *r*_*li*_ denotes the rate constant for autolysosome formation from Si with appropriate constants *k*_*l*_ and *p*_*l*_ for ATP, based on biological experiments [40, 41].

The intralysosomal hydrolysis specific rate *R*_*hi*_ is taken as a function of the intracellular ATP concentration (*i* = 1, 2, and 3):

13 $$ {R}_{hi}\left({C}_{\mathrm{A}}\right)={r}_{hi}\frac{{C_{\mathrm{A}}}^{\delta_h}}{{C_{\mathrm{A}}}^{\delta_h}+{k_h}^{\delta_h}}, $$

with appropriate exponent *δ*_*h*_ and constant *k*_*h*_ for ATP, where *r*_*hi*_ is the rate constant for intralysosomal hydrolysis [40, 41]. Further details of the equations for autolysosome formation and intralysosomal hydrolysis can be found in literature [4, 9, 49, 50].

#### Secretion and clearance of amyloid-β

Considering that Aβ secretion from the intra to extra cellular space of a neuron is positively correlated with the autophagy induction level [29–31], we assume the Aβ secretion specific rate *R*_*sec*_ to be proportional to the degree of amino acid- and ATP-dependent autophagosome induction, as defined in Eqs. (9)–(11), with an appropriate constant *r*_*sec*_:

14 $$ {R}_{sec}\left({C}_{\mathrm{a}},{C}_{\mathrm{A}}\right)={r}_{sec}\frac{{C_{\mathrm{A}}}^4}{{C_{\mathrm{A}}}^4+{k_g}^4}\frac{{p_g}^{12}}{{C_{\mathrm{A}}}^{12}+{p_g}^{12}}\left(1+{\gamma}_g{e}^{-{\xi}_g{C}_{\mathrm{a}}}\right). $$

The concentration-dependent biphasic Aβ clearance rate *R*_*clr*_ in the extracellular space is assumed, on the basis of biological experiments [35, 36, 51], to take the form:

15 $$ {R}_{clr}\left({C}_{\mathrm{ES}3}\right)={r}_{clr}\left({C}_{\mathrm{ES}3}+{\omega}_{ext}\right), $$

where *r*_*clr*_ denotes the rate constant for Aβ clearance, with an appropriate constant *ω*_*ext*_. The rate of Aβ clearance varies with the concentration according to the measurement on Alzheimer’s mouse model [35]: While the half-life is very short at high concentrations of extracellular Aβ, it grows longer as the concentration decreases. Equation (15) captures qualitatively this biphasic nature of Aβ clearance [35] and its value lies within a reasonable range consistent with the state-of-the-art measurements [36, 51].

#### Protein synthesis and non-autophagic degradation

The (total) protein synthesis rate *R*_*S*_ which depends on intracellular concentrations *C*_a_ of amino acids and *C*_A_ of ATP reads [52].

16 $$ {R}_S\left({C}_{\mathrm{a}},{C}_{\mathrm{A}}\right)=\Big\{{\displaystyle \begin{array}{c}{r}_s\frac{C_{\mathrm{a}}}{C_{\mathrm{a}}+{k}_s}\frac{\exp \left[{C}_{\mathrm{A}}\right]-1}{\exp \left[{C}_{\mathrm{A}}^{(m)}\right]-1}\kern1em \mathrm{for}\ {C}_{\mathrm{A}}<{C}_{\mathrm{A}}^{(m)}\\ {}{r}_s\frac{C_{\mathrm{a}}}{C_{\mathrm{a}}+{k}_s}\kern6em \mathrm{for}\ {C}_{\mathrm{A}}\ge {C}_{\mathrm{A}}^{(m)}\end{array}} $$

with appropriate constant *k*_*s*_ for amino acid, where ${C}_{\mathrm{A}}^{(m)}$ is the ATP concentration corresponding to the maximal protein synthesis rate and *r*_*s*_ denotes the rate constant for the protein synthesis. Further details of the protein synthesis can be found in literature [4, 9, 49, 50].

The non-autophagic protein degradation machinery such as the ubiquitin-proteasome system has been considered in the model. We assume that the amount of protein degradation by autophagy constitutes 80% of the total amount of protein degradation and the non-autophagic protein degradation machinery is responsible for the remaining 20% [53]. Accordingly, we take the rate of non-autophagic degradation *R*_*di*_ (*i* = 1, 2, and 3) to be 25% of autophagic degradation:

17 $$ {R}_{di}=\frac{1}{4}{R}_{hi}{C}_{\mathrm{li}}. $$

## Results

### Aβ kinetics under normal and pathological conditions

**Fig. 2:**
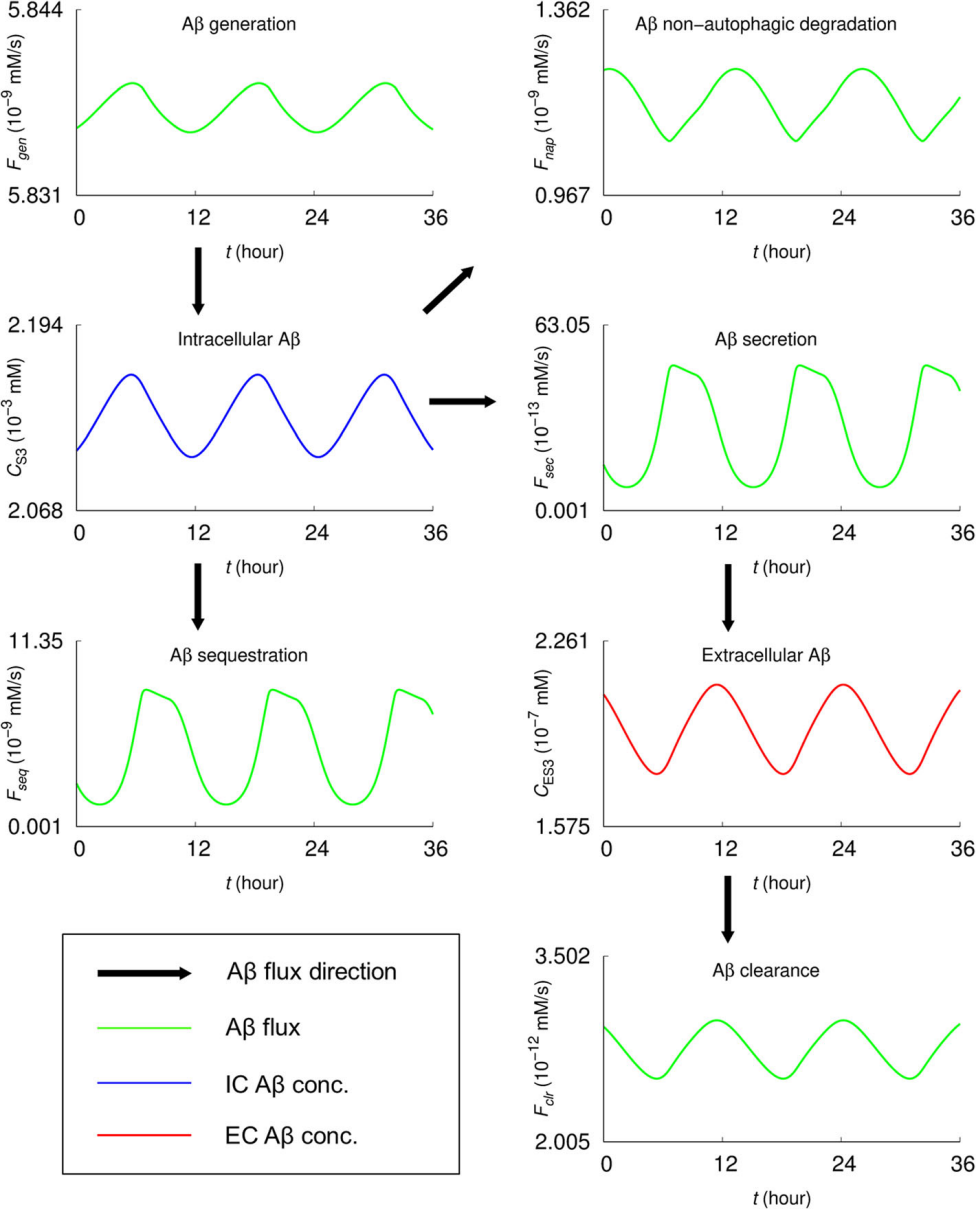
The basal steady-state Aβ concentrations and fluxes. *C*_S3_ and *C*_ES3_ represent the concentrations of intracellular (IC) and extracellular (EC) Aβ, respectively. *F*_*gen*_, *F*_*seq*_, and *F*_*nap*_ denote the Aβ generation, sequestration (the first step of autophagic degradation, i.e., autophagosome formation), and non-autophagic degradation fluxes, respectively; *F*_*sec*_ and *F*_*clr*_ the secretion (from the IC to the EC space) and Aβ clearance (in the EC space) fluxes, respectively

In Fig. 2, the relation of intracellular (*C*_S3_) and extracellular (*C*_ES3_) Aβ levels with the respective Aβ fluxes under normal conditions (i.e., for basal parameter values) are shown, providing kinetic and dynamic insights into the Aβ regulation. As illustrated in Fig. 1, *C*_S3_ (the second row of the first column) is determined by the difference between influx (i.e., Aβ generation flux, denoted by *F*_*gen*_, the concentration of Aβ generated per unit time given in units of mM/s) and efflux rates such as autophagic sequestration *F*_*seq*_ (the concentration of intracellular Aβ sequestered into autophagosomes per unit time, i.e., *F*_*seq*_ *= R*_*g*3_*C*_S3_), non-autophagic degradation *F*_*nap*_ (the concentration of intracellular Aβ degraded via the non-autophagic mechanism per unit time, i.e., *F*_*nap*_ *= R*_*d*3_), and secretion *F*_*sec*_ (the concentration of intracellular Aβ secreted from the inside to outside of a neuron per unit time, i.e., *F*_*sec*_ = *R*_*sec*_*C*_S3_). *C*_ES3_ (the third row of the second column) is governed by *F*_*sec*_ and the clearance flux *F*_*clr*_ (the concentration of Aβ removed from the extracellular space per unit time, i.e., *F*_*clr*_ *= R*_*clr*_*C*_ES3_).

**Fig. 3:**
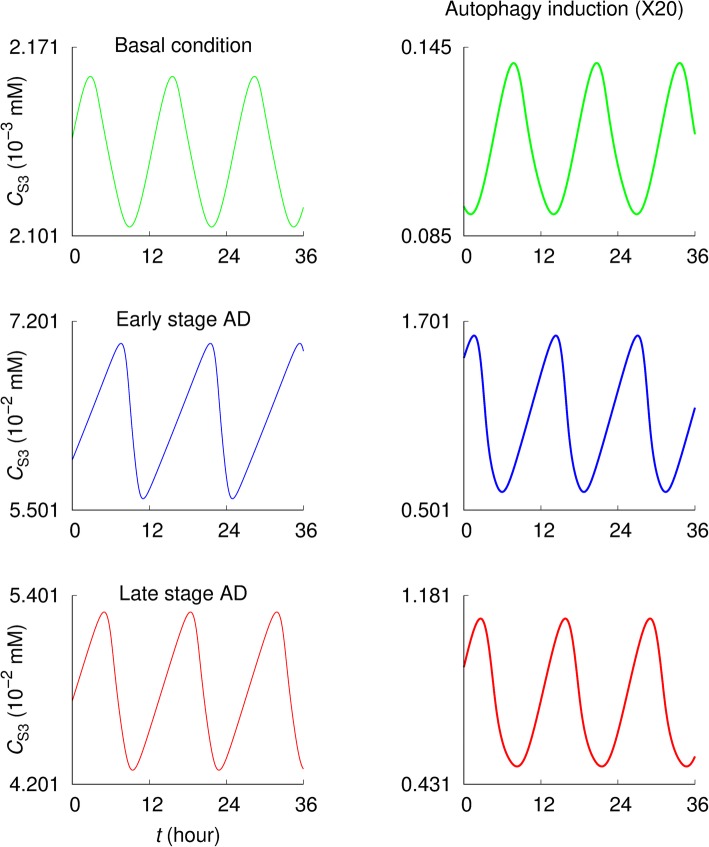
Intracellular Aβ concentrations under normal and pathological conditions. The intracellular Aβ concentration *C*_S3_ displays oscillatory behaviors depending on the parameters. The basal value of Aβ generation rate constant (i.e., *β = β*^(0)^) has been used for the normal condition while *β* = 100 × *β*^(0)^ has been used for the early stage AD. For the late stage AD, the specific rate constants of autolysosome formation and intralysosomal hydrolysis have been set equal to *r*_*l*3_ = 0.1 × *r*_*l*3_^(0)^ and *r*_*h*3_ = 0.1 × *r*_*h*3_^(0)^, retaining the high Aβ generation rate as the early state AD. The results in the second column were obtained under 20-fold increase in the autophagosome formation rate constant (*r*_*g*3_ = 20 × *r*_*g*3_^(0)^) with others the same as those in the first column

**Fig. 4:**
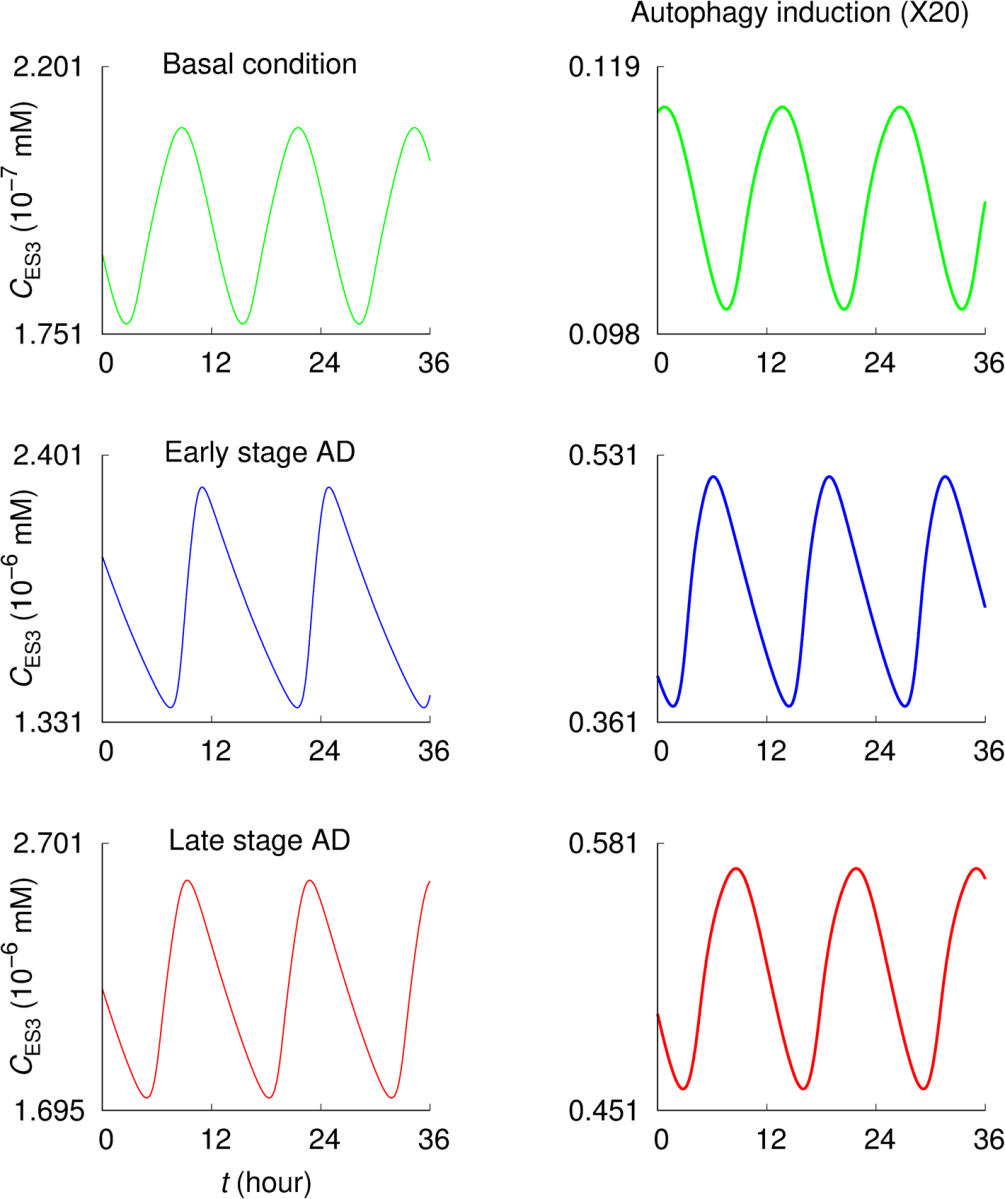
Extracellular Aβ concentrations under normal and pathological conditions. The extracellular Aβ concentration *C*_ES3_ displays oscillatory behaviors depending on the parameters. The basal value of the Aβ generation rate constant (*β = β*^(0)^) was used for the normal condition while a high Aβ generation rate *β* = 100 × *β*^(0)^ has been used for the early and late stage AD. For the late stage AD, the specific rate constants of autolysosome formation and intralysosomal hydrolysis have been set equal to *r*_*l*3_ = 0.1 × *r*_*l*3_^(0)^ and *r*_*h*3_ = 0.1 × *r*_*h*3_^(0)^, in addition to the high Aβ generation rate. The results in the second column were obtained under 20-fold increase in the autophagosome formation rate constant (*r*_*g*3_ = 20 × *r*_*g*3_^(0)^), with other parameters remaining unchanged

Figures 3 and 4 compare values of *C*_S3_ and *C*_ES3_, respectively, under the normal, early stage (i.e., abnormal increase in Aβ generation), and late stage AD (i.e., increased Aβ generation together with decreased autophagic lysosomal degradation) conditions [28]. The simulations have been performed with the basal value *β*^(0)^ of the Aβ generation rate constant, i.e., *β = β*^(0)^, for the normal condition, while data for the early and late stage AD conditions have been obtained at an extremely high Aβ generation rate, *β =* 100 × *β*^(0)^. Further, in the late stage case, the specific rate constants of autolysosome formation and intralysosomal hydrolysis have been set to be 10% of the basal values, i.e., *r*_*l*3_ = 0.1 × *r*_*l*3_^(0)^ and *r*_*h*3_ = 0.1 × *r*_*h*3_^(0)^.

It is observed that *C*_S3_ and *C*_ES3_ are significantly higher in AD conditions than in the basal condition—*C*_S3_ is higher at the early stage than at the late stage AD (Fig. 3) while *C*_ES3_ is higher at the late stage AD (Fig. 4). In both pathological conditions, autophagy induction (i.e., a 20-fold increase in the autophagosome formation rate constant: *r*_*g*3_ = 20 × *r*_*g*3_^(0)^) significantly reduces *C*_S3_ and *C*_ES3_. In addition, the early and late stage AD exhibit asymmetric oscillating patterns. *C*_S3_ increases gradually and then drops rapidly; conversely, *C*_ES3_ increases rapidly and drops gradually. Under the basal condition they exhibit relatively symmetrical oscillation patterns.

**Fig. 5:**
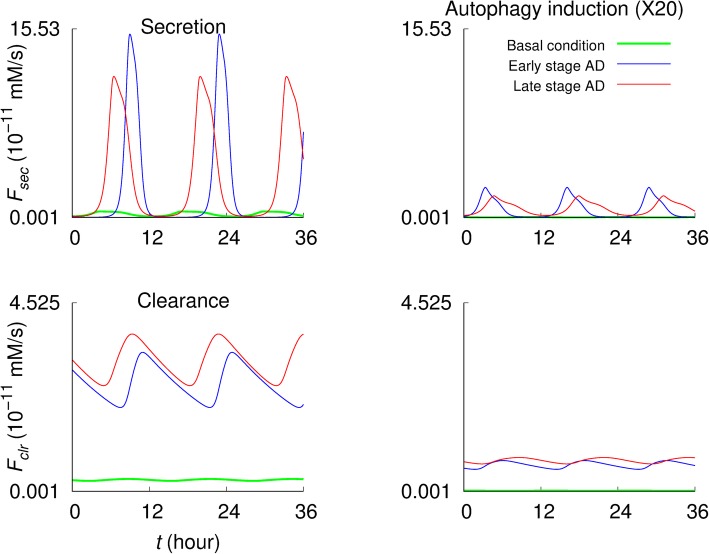
Aβ secretion and clearance fluxes in normal and pathological conditions. *F*_*sec*_ and *F*_*clr*_ denote the Aβ secretion flux (from the intracellular to the extracellular space) and Aβ clearance flux in the extracellular space, respectively. The results in the second column have been obtained under 20-fold increase in the autophagosome formation rate constant (*r*_*g*3_ = 20 × *r*_*g*3_^(0)^), with other parameters kept unchanged

Both Aβ secretion flux *F*_*sec*_ and clearance flux *F*_*clr*_ are significantly promoted in the early and late stage AD cases compared to those in the basal condition (the first column of Fig. 5). The peaks of *F*_*sec*_ in early AD are sharper and higher but stay at the near-zero rate for a longer period than in late AD. In contrast, *F*_*clr*_ exhibits higher peaks in late AD than in early AD. Autophagy induction (i.e., *r*_*g*3_ = 20 × *r*_*g*3_^(0)^) significantly reduces those fluxes, close to the basal levels.

In what follows, autophagy dynamics corresponding to the normal and AD conditions are presented, including steady-state concentrations of autophagosome, autolysosome, and autophagic fluxes.

### Dynamics of autophagy and implications in the Aβ regulations

**Fig. 6:**
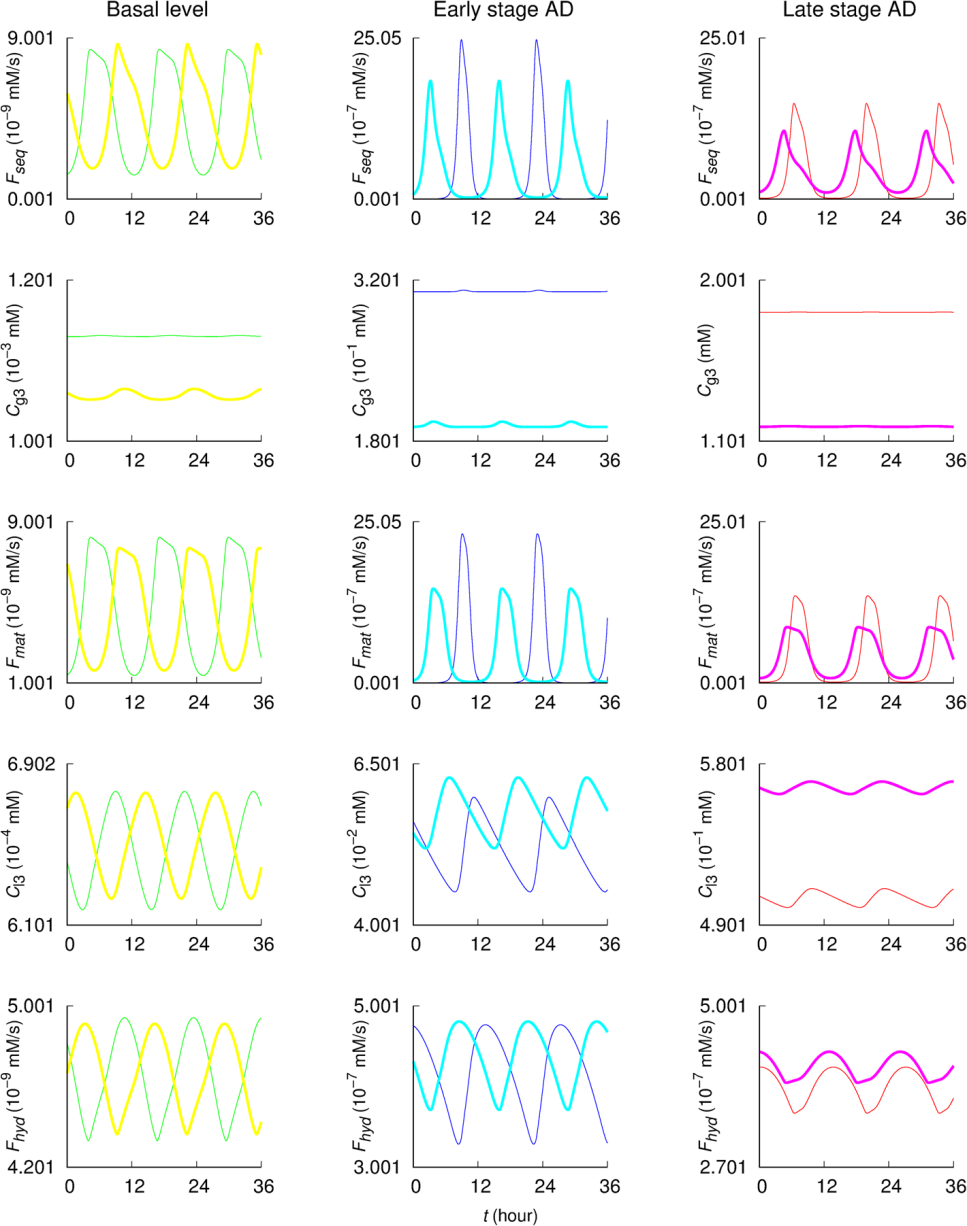
Dynamics of autophagy. *F*_*seq*_, *F*_*mat*_, and *F*_*hyd*_ denote fluxes of protein sequestration (i.e., autophagosome formation), autophagosome maturation (i.e., autolysosome formation), and intralysosomal hydrolysis steps, respectively. *C*_g3_ and *C*_l3_ are the autophasosome and autolysosome concentrations in Aβ, respectively. Yellow, cyan, and purple lines plot results of autophagy induction (i.e., *r*_*g*3_ = 20 × *r*_*g*3_^(0)^) in the cases of the basal condition, early stage AD, and late stage AD, respectively. Green, blue, and red lines plot results from simulations with *r*_*g*3_^(0)^ in the same three cases (basal, early stage AD, and late stage AD), respectively

Protein sequestration (i.e., autophagosome formation) flux *F*_*seq*_, autophagosome maturation (i.e., autolysosome formation) flux *F*_*mat*_, and intralysosomal hydrolysis flux *F*_*hyd*_ in both early and late stage AD are significantly increased compared with those on the basal condition (the first, third, and fifth rows of Fig. 6). The peaks of *F*_*seq*_ and *F*_*mat*_ in early stage AD are sharper and higher than those in the late stage. The steady-state concentrations of autophagosomes and autolysosomes, *C*_g3_ and *C*_l3_, in the AD cases are greater than those in the basal condition case: the values at the late stage of AD are about ten times greater than those at the early stage (the second and fourth rows of Fig. 6).

In the cases of early and late stage AD, autophagy induction (i.e., *r*_*g*3_ = 20 × *r*_*g*3_^(0)^) significantly decreases *F*_*seq*_ and *F*_*mat*_, while it increases *F*_*hyd*_ (the first, third, and fifth rows of the second and third columns of Fig. 6). The steady-state autophagosome concentration *C*_g3_ is decreased while the autolysosome concentration *C*_l3_ is increased upon autophagy induction (the second and fourth rows of the second and third columns of Fig. 6). Under the basal condition, the oscillatory patterns of autophagic fluxes and steady-state concentrations of autophagosomes and autolysosomes are not significantly affected by the autophagy induction, compared to the AD cases.

As shown above, autophagy induction (i.e., ${r}_{g3}=20\times {r}_{g3}^{(0)}$) significantly reduces *C*_S3_ and *C*_ES3_. Increasing *r*_*g*3_ beyond $20\times {r}_{g3}^{(0)}$ reduces the Aβ levels further, until they reach basal levels. However, the required value of *r*_*g*3_ to bring the basal levels may vary depending on the stage of AD and the activities of the other autophagic steps such as autophagosome maturation (i.e., autolysosome formation) and intralysosomal hydrolysis.

**Fig. 7:**
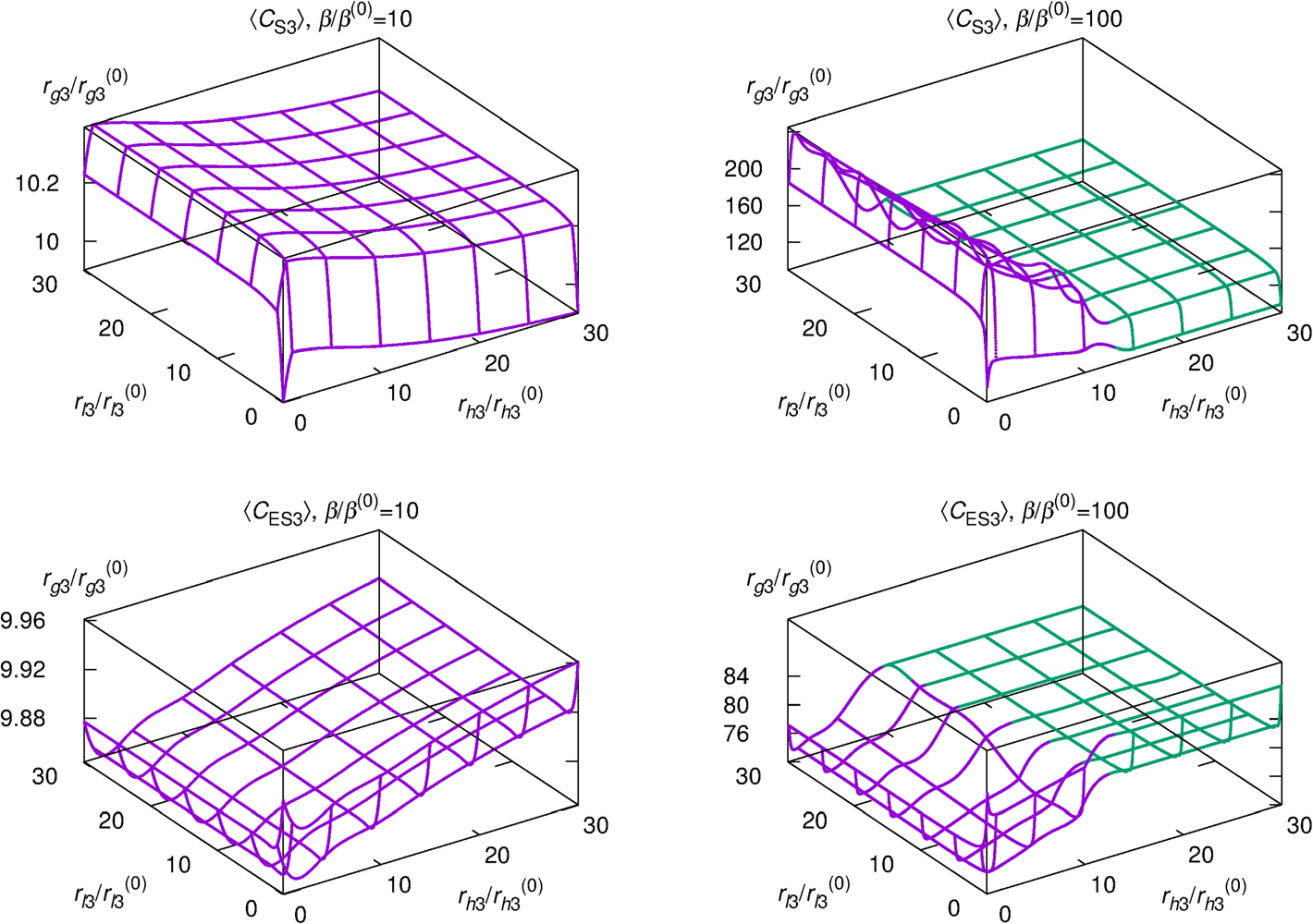
Aβ concentrations depending upon activities of three autophagy steps. The surfaces specify time-averaged intracellular Aβ concentration 〈*C*_S3_〉 (first row) and extracellular Aβ concentration 〈*C*_ES3_〉 (second row) for basal parameter values; regions above and below the surfaces correspond to Aβ concentrations lower and higher than the basal values. The first and the second columns correspond to *β/β*^(0)^ *=* 10 and *β/β*^(0)^ *=* 100, respectively. Computations were performed with *r*_*l*3_/*r*_*l*3_^(0)^ and *r*_*h*3_/*r*_*h*3_^(0)^ varied in increments and the mixed cubic and quintic spline interpolation applied. On the surfaces in purple the Aβ concentrations display oscillations while oscillations are absent on the green surfaces

Figure 7 presents a three-dimensional surface plot, exhibiting step-specific and combined effects of the autophagy pathway on Aβ levels for a moderately high Aβ formation rate *β*/*β*^(0)^ = 10 (the first column) and an extremely high formation rate *β*/*β*^(0)^ = 100 (the second column). The vertical axis measures the autophagosome formation rate relative to its normal value (i.e., *r*_*g*3_*/r*_*g*3_^(0)^) and the two horizontally placed axes represent the autolysosome formation and the intralysosomal hydrolysis rates relative to the normal values, spanning the range from highly induced activity (*r*_*l*3_/*r*_*l*3_^(0)^ = *r*_*h*3_/*r*_*h*3_^(0)^ = 30) to normal (*r*_*l*3_/*r*_*l*3_^(0)^ = *r*_*h*3_/*r*_*h*3_^(0)^ = 1) and extremely reduced activity (*r*_*l*3_/*r*_*l*3_^(0)^ = *r*_*h*3_/*r*_*h*3_^(0)^ = 0.1). The surfaces designate time-averaged intracellular Aβ concentration 〈*C*_S3_〉 (top) and extracellular Aβ concentration 〈*C*_ES3_〉 (bottom) for basal parameter values (i.e., under normal conditions); regions above and below the surface correspond to Aβ concentrations lower and higher than the basal values, respectively.

**Fig. 8:**
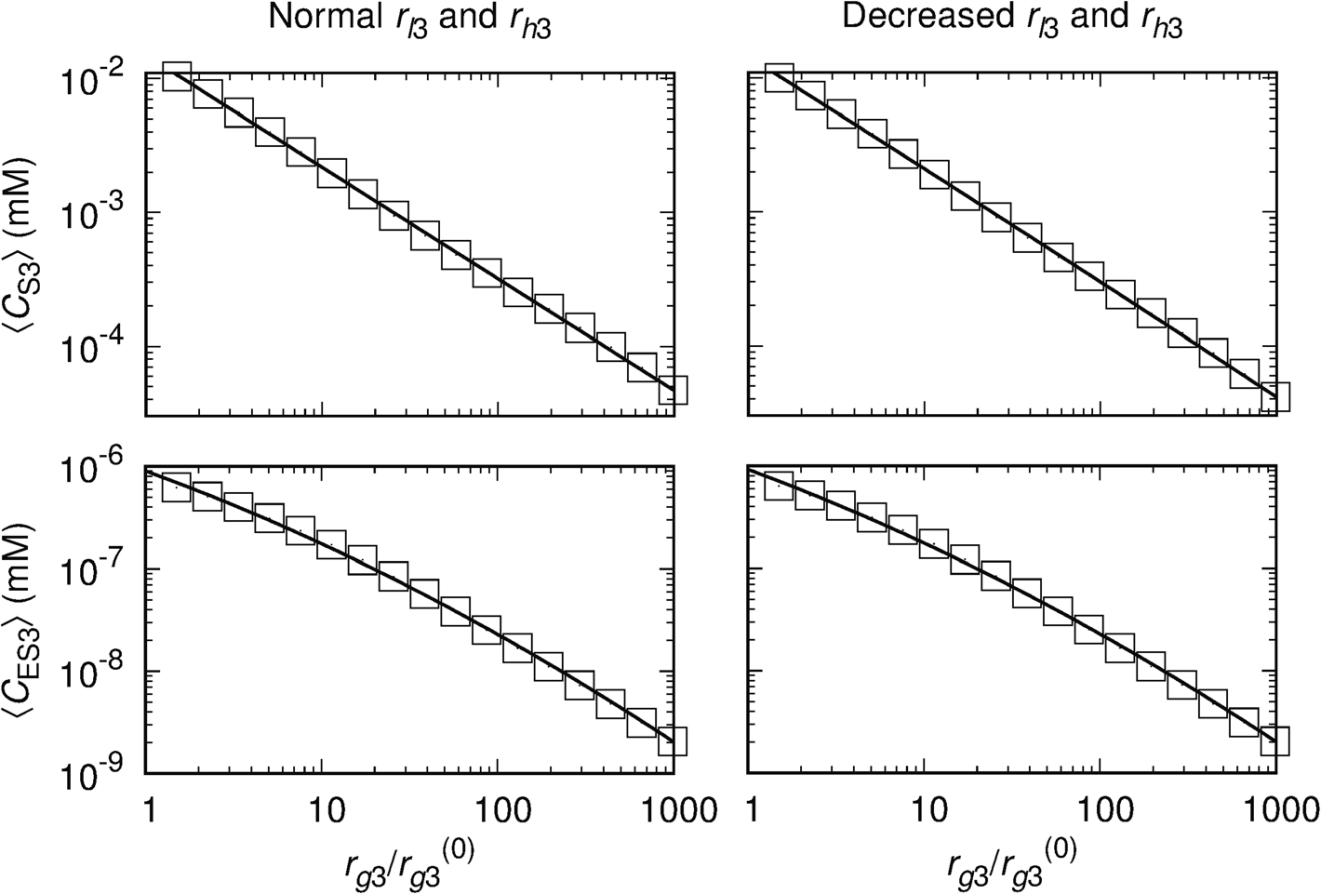
Log-normal relations between average Aβ concentrations and *r*_*g*3_*/r*_*g*3_^(0)^. Log-log plots of 〈*C*_S3_〉 (top) and 〈*C*_ES3_〉 (bottom) versus *r*_*g*3_*/r*_*g*3_^(0)^ for *r*_*l*3_/*r*_*l*3_^(0)^ = *r*_*h*3_/*r*_*h*3_^(0)^ = 1 (left column) and 0.1 (right column). Data were obtained at *β/β*^(0)^ *=* 10. Squares indicate average values obtained via simulations and lines depict the least square fit of the log-normal relation

For both Aβ synthesis rates (*β*/*β*^(0)^ = 10 and 100), 〈*C*_S3_〉 and 〈*C*_ES3_〉 decrease with *r*_*g*3_ in a log-normal manner, ${\left\langle C\right\rangle}_{r_{g3}/{r}_{g3}^{(0)}=x}=\left(\frac{\gamma }{x\sigma \sqrt{2\pi }}\right)\exp \left[-{\left(\log\ x-\mu \right)}^2/2{\sigma}^2\right]$, where 〈*C*〉 denotes 〈*C*_S3_〉 or 〈*C*_ES3_〉 and *γ*, *σ*, and *μ* are adjustable parameters (Fig. 8). When *r*_*l*3_ is decreased from 1 to 0.1, 〈*C*_S3_〉 decreases while 〈*C*_ES3_〉 increases. In contrast, when *r*_*l*3_ > 1, the concentrations are relatively independent of *r*_*l*3_. The effects of *r*_*h*3_ generally follow the trend.

**Fig. 9:**
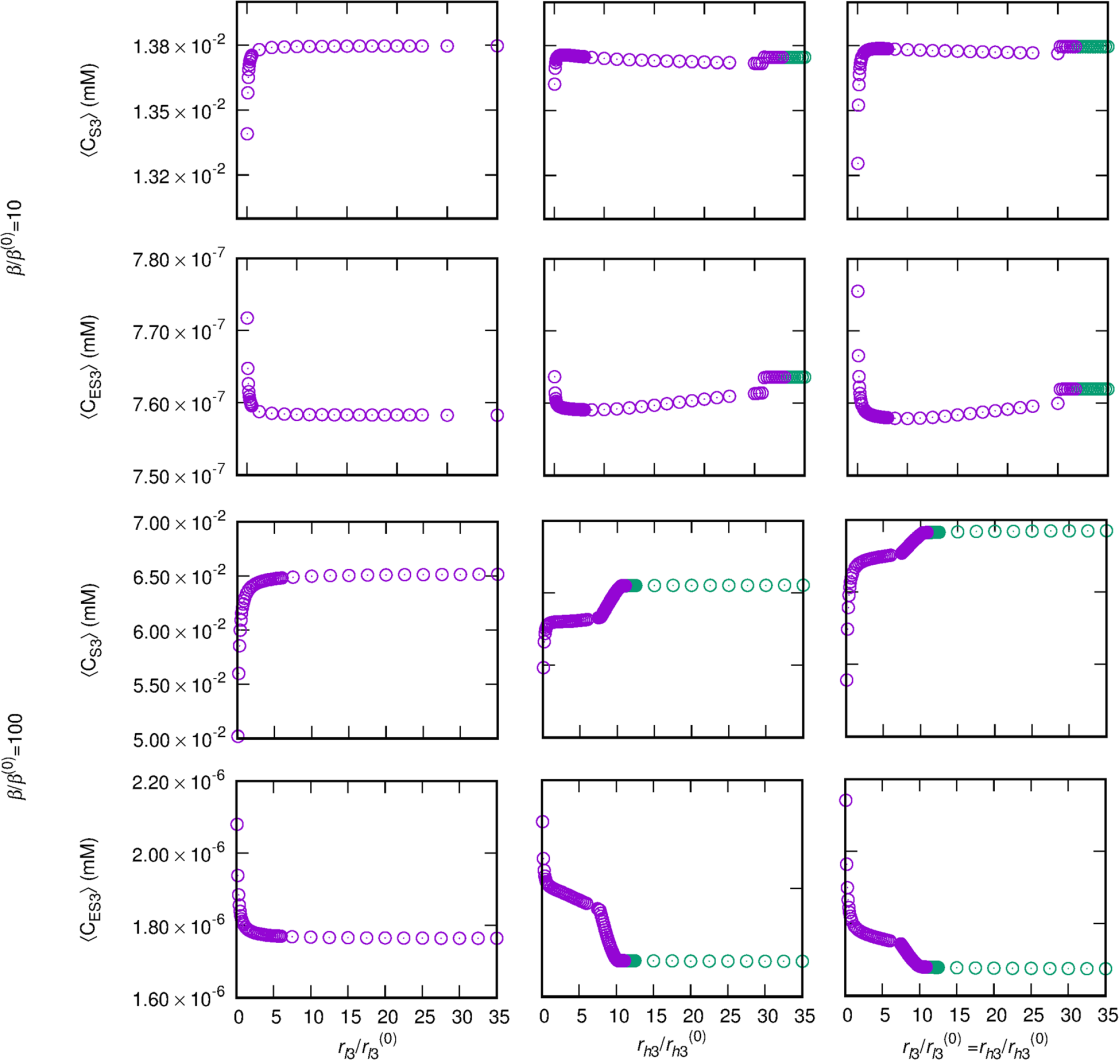
Effects of *r*_*l*3_ and *r*_*h*3_ on Aβ concentrations. Average intracellular Aβ concentration 〈*C*_S3_〉 (first and third rows) and extracellular Aβ concentration 〈*C*_ES3_〉 (second and fourth rows) at *β/β*^(0)^ *=* 10 (upper two rows) and *β/β*^(0)^ *=* 100 (lower two rows), depending upon changes of *r*_*l*3_*/r*_*l*3_^(0)^ (first column), *r*_*h*3_*/r*_*h*3_^(0)^ (second column), and *r*_*l*3_*/r*_*l*3_^(0)^ and *r*_*h*3_*/r*_*h*3_^(0)^ together (third column). At data points in purple, oscillations of Aβ concentrations are observed; at green data points, concentrations are stationary

The surface shape of Fig. 7 reflects the combined effects of the three-autophagy steps. A greater vale of *r*_*g*3_ is required to return to basal values in the case *β*/*β*^(0)^ = 100 compared with the case *β*/*β*^(0)^ = 10. At *r*_*l*3_/*r*_*l*3_^(0)^ < 1 and *r*_*h*3_/*r*_*h*3_^(0)^ < 1 both concentrations change greatly compared with the case *r*_*l*3_/*r*_*l*3_^(0)^ > 1 and *r*_*h*3_/*r*_*h*3_^(0)^ > 1, indicating that reduction of autolysosome formation and/or intralysosomal hydrolysis has greater impact on the Aβ concentrations than promotion of these steps. Above *r*_*h*3_*/r*_*h*3_^(0)^ = ~ 45.2 (for *β*/*β*^(0)^ = 10) and *r*_*h*3_*/r*_*h*3_^(0)^ = ~ 11.1 (for *β*/*β*^(0)^ = 100), the oscillations of proteins (*C*_S1_, *C*_S2_, *C*_S3_, and *C*_ES3_), ATP (*C*_A_), and amino acids (*C*_a_) disappear, converging to stationary values (green surfaces in Figs. 7 and 9). In the stationary region, the effects of *r*_*l*3_/*r*_*l*3_^(0)^ and *r*_*h*3_/*r*_*h*3_^(0)^ are minimal, as manifested by the flatness of the green surface.

## Discussion

In this study we have investigated via modeling and simulations how autophagy activity affects Aβ kinetics such as the intra and extracellular levels, secretion, clearance, and autophagic degradation. The mathematical model has been extended from the multi-compartment autophagy model originally developed by Han and Choi [4, 9, 49, 50] to the one with Aβ kinetics incorporated by accommodating the current working hypothesis [29–31] and the experimental mechanistic studies [28–36, 51] on the relationship between autophagy activity and Aβ kinetics. Such multi-compartment frameworks [4, 9, 49, 50] are especially useful for testing biological hypotheses regarding the selective autophagy including Aggrephagy (i.e., autophagic degradation of protein aggregates), Mitophagy (for mitochondria), and Xenophagy (for microbes) [54] because the model can be easily modified easily to incorporate new substrates for selective degradation in each compartment (see Fig. 1). This approach can be further improved by including detailed mathematical descriptions of autophagy-related cellular signaling pathways, which have been extensively explored in recent years [55–59].

The analysis began with the profiles of Aβ fluxes governing the intracellular and extracellular Aβ concentrations under the normal conditions. As shown in Fig. 2, the intracellular Aβ concentration is determined by the difference between influx (i.e., Aβ generation flux) and efflux rates of autophagic sequestration, non-autophagic degradation, and Aβ secretion, while the extracellular Aβ concentration is governed by Aβ secretion and clearance. This provides an overview of the system—how the Aβ levels might be determined, giving the idea of how to maintain normal Aβ levels against pathological conditions. Promoting autophagic sequestration flux (i.e., autophagy induction) would significantly reduce the intracellular and extracellular Aβ concentrations for the early and the late stage AD (Figs. 3 and 4). Interestingly, the intracellular concentration is higher in early stage than late stage AD, while extracellular concentration is higher in late stage AD. Aβ secretion and clearance fluxes are promoted in the early and late stage AD compared to the normal condition (Fig. 5). In both pathological conditions, promoting autophagic sequestration efficiently decreases the Aβ secretion and clearance fluxes.

In the examination of autophagy dynamics under normal and pathological conditions (Fig. 6), the autophagic fluxes and the concentrations of autophagosome (*C*_g3_) and autolysosome (*C*_l3_) in both early and late stage AD are significantly increased than in the basal condition. *C*_g3_ and *C*_l3_ are about ten times greater in late stage AD than in early stage AD. This implies that at the late stage AD the increased concentrations due to reduced maturation and intralysosomal hydrolysis may clog neurons, which would further reduce the autophagic Aβ degradation efficacy. Under normal conditions the basal autophagy level is sufficient for removing intracellular Aβ as the mTOR activity is tightly regulated. However, during early and late stage of AD, an increase in soluble Aβ levels leads to mTOR hyperactivity, which should in turn suppress autophagosome formation (i.e., reduced Aβ sequestration) (for details see ***Autophagosome formation*** in **Mathematical model**). Reduced autophagosome formation would increase further the Aβ levels, creating a vicious cycle.

The influence of each autophagic step on the intracellular and the extracellular Aβ concentrations (*C*_S3_ and *C*_ES3_) was examined, providing insight into disease and potential effects of drugs targeting specific steps in the autophagic pathway. The autophagosome formation activity plays a significant role in regulating average values of *C*_S3_ and *C*_ES3_ via a log-normal relation: promoting the autophagosome formation step decreases both Aβ levels. As the autolysosome formation and intralysosomal hydrolysis rates are decreased, as expected in late stage AD, *C*_S3_ decreases but *C*_ES3_ increases. It is thus disclosed that the progress from early to late stage AD leads to higher *C*_ES3_ levels, which could contribute to the deposition of extracellular plaques. On the other hand, *C*_S3_ decreases along the pathway to late stage AD (i.e., autophagic Aβ degradation is defective in addition to the increased Aβ generation).

The model has reproduced successfully the oscillatory behavior of autophagy activity concerning the autophagy-related fluxes and the concentrations of Aβ, autophagosomes, and autolysosomes (Figs. 2-6). Such simulated “autophagy oscillations” are qualitatively similar to those observed in biological experiments [60–69]. However, mechanisms underlying the phenomena have only begun to be explored [68–70]. For instance, the oscillations might be tightly controlled via the autophagy-related signaling pathways to keep the autophagy activity within physiological levels that is important for cellular homeostasis. The simulation results presented here exhibit two interesting features: 1) In the early- and late-stage AD, oscillations of C_S3_ and C_ES3_ exhibit asymmetric patterns while they are symmetric under the basal condition. 2) Above certain activity levels of autolysosome formation (measured by *r*_*l*3_) and intralysosomal hydrolysis (*r*_*h*3_) for Aβ, there disappear oscillations of proteins (C_S1_, C_S2_, C_S3_, and C_ES3_), ATP (C_A_), and amino acids (C_a_).

These findings are expected to be useful for the design of future studies and may give insight to maintaining physiological regulation of the Aβ levels. Defects arising in different steps of the autophagy process would influence in a different way the Aβ kinetics, which will give rise to distinct AD pathology. This suggests that pharmacological modulations of the different autophagy steps may have different implications for AD therapy and prevention.

## Conclusions

A mathematical model of autophagy and Aβ metabolism has been developed by integrating experimental knowledge of individual mechanisms. It has been observed that the different steps of the autophagy pathway have different effects on the Aβ levels. Promotion of Aβ sequestration has led to a reduction of both intracellular and extracellular Aβ, while suppression of autophagosome maturation and intralysosomal hydrolysis has had opposing effects, increasing intracellular and decreasing extracellular Aβ. The model thus predicts that modulations of different steps have significant step-specific and combined effects on Aβ levels, suggesting therapeutic and preventive implications of autophagy on AD.

## Methods

A mathematical model is developed to examine roles of autophagy in modulating Aβ kinetics. The model includes a nonlinear relationship between autophagy activity and intracellular and extracellular Aβ levels. Autophagy degrades intracellular Aβ and influences the Aβ secretion from the inside to the outside of the neuron (i.e., extracellular space) and the concentration-dependent biphasic Aβ clearance in the extracellular space. Conversely, the intracellular Aβ level regulates the autophagy induction step (i.e., autophagosome formation or protein sequestration). The dynamics of these relations are described by twelve coupled differential equations which are solved via the 5th order Runge-Kutta method for very high precision. Mixed spline interpolation has been used to produce the three-dimensional surface plots of the Aβ concentrations.

## Data Availability

Not applicable.
